# Association Between the Severity of Periodontitis and Temporomandibular Joint Symptoms in Patients Requiring Prosthodontic Rehabilitation: A Cross-Sectional Study

**DOI:** 10.7759/cureus.86493

**Published:** 2025-06-21

**Authors:** Tripti Bhoi, Nosheen Riza, Harshita Pandey, Sumaiya Iman, Premraj Jadhav, Rupali Malik, Seema Gupta

**Affiliations:** 1 Department of Oral and Maxillofacial Pathology, D Y Patil Dental School, Pune, IND; 2 Department of Periodontology, New Horizon Dental College and Research Institute, Bilaspur, IND; 3 Department of Public Health Dentistry, Teerthanker Mahaveer Dental College and Research Centre, Moradabad, IND; 4 Department of Prosthodontics, Kothiwal Dental College and Research Centre, Moradabad, IND; 5 Department of Prosthodontics, Yogita Dental College and Hospital, Khed, IND; 6 Department of Orthodontics, Kothiwal Dental College and Research Centre, Moradabad, IND

**Keywords:** pain, periodontitis, principal component analysis, prosthodontic, temporomandibular joint

## Abstract

Introduction

Periodontitis and temporomandibular joint (TMJ) disorders are prevalent conditions that may influence prosthodontic treatment outcomes, owing to their impact on occlusal stability and jaw function. Understanding this association is critical for optimizing treatment planning and improving patient outcomes. This study investigated the relationship between the severity of periodontitis and clinical TMJ symptoms in patients requiring prosthodontic restoration, with the aim of providing evidence-based guidance for integrated clinical management.

Materials and methods

A cross-sectional observational study was conducted on 80 adult patients (aged 18-55 years) with different stages of periodontitis and a clinical indication for prosthodontic rehabilitation, excluding those with systemic diseases affecting the TMJ or prior TMJ surgery. Periodontal status was assessed using probing depth, clinical attachment loss, and cone-beam computed tomography (CBCT) to evaluate condylar morphology and the articular eminence angle. TMJ symptoms were evaluated through clinical examination of jaw movement, joint sounds, and tenderness. The Fonseca Anamnestic Index (FAI) was used to assess the presence and intensity of symptoms of temporomandibular joint disorders (TMDs). Prosthodontic treatment plans and occlusal characteristics were documented. Data were analyzed using the chi-square test, the Kruskal-Wallis test, and principal component analysis (PCA), with a significance level of p < 0.05.

Results

Significant associations were found between the severity of periodontitis and TMJ symptoms, particularly tenderness on palpation and changes in condylar morphology. Functional impairments, including reduced mouth opening and increased deviation, worsened with the severity of periodontitis. The FAI, which reflects pain and dysfunction, progressively escalated across the periodontal groups. Structural changes, such as steeper articular eminence inclination, were prominent in patients with severe periodontitis. PCA identified a TMJ pathology continuum driven by periodontitis severity.

Conclusion

The severity of periodontitis was correlated with worsened TMJ symptoms, impacting prosthodontic treatment planning. Integrated periodontal and TMJ management is essential for optimizing functional restoration and patient comfort during prosthodontic care.

## Introduction

Periodontitis is a prevalent inflammatory condition characterized by progressive destruction of periodontal tissues, including the gingiva, periodontal ligament, and alveolar bone, ultimately leading to tooth loss if untreated [1]. Ramfjord et al. [2], in their study, analyzed a World Health Organization (WHO) survey conducted in India, along with four additional nations. They reported a 100% prevalence of periodontal disease (including gingivitis) in India. The onset of periodontitis was identified to commence after the age of 15 years; by the age of 17 years, 10% of Indian males exhibited signs of periodontitis. The disease is primarily driven by bacterial biofilms, which trigger a host inflammatory response and cause clinical manifestations such as gingival bleeding, periodontal pocketing, and tooth mobility [1].

Temporomandibular joint disorders (TMDs), on the other hand, encompass a spectrum of neuromuscular and musculoskeletal conditions affecting the temporomandibular joint (TMJ), masticatory muscles, and associated structures, often presenting with symptoms like jaw pain, restricted movement, and joint sounds [3]. Both conditions are significant in dental practice, particularly in patients requiring prosthodontic restoration, in whom occlusal stability and functional rehabilitation are critical [4].

Emerging research suggests a complex interplay between periodontitis and TMD, particularly in patients requiring prosthodontic interventions [5,6]. Periodontitis can lead to unilateral mastication due to discomfort or tooth loss, which may induce biomechanical stress on the TMJ, potentially causing pain and structural changes [5]. For instance, Jeon et al. [5] found that unilateral chewing, resulting from periodontitis, was associated with increased pain and altered TMJ morphology, emphasizing the need for timely periodontal intervention to mitigate secondary TMJ issues. Based on a retrospective analysis involving 4,204 randomly chosen subjects, noteworthy correlations were established between missing teeth and challenges in mastication [7], which adversely affected the TMJ. An increase in mechanical stress or microtrauma may precipitate TMDs in edentulous areas. Moreover, these conditions are associated with complex biological mechanisms, including stimulation of inflammatory responses and immune system activation, in addition to degradation of constituents within the extracellular matrix [8]. The bidirectional relationship between periodontitis and TMD is complicated in prosthodontic patients, where occlusal discrepancies or restorations may exacerbate TMD symptoms, or may be influenced by periodontal health [5].

Prosthodontic treatment aims to restore occlusal stability and function, often involving crowns, bridges, or implants, but must account for underlying periodontal and TMJ conditions to ensure long-term success [9]. Patients with severe periodontitis may exhibit compromised periodontal support, which can affect prosthetic planning and outcome. Similarly, TMD symptoms, such as muscle pain or joint dysfunction, may necessitate a multidisciplinary approach involving periodontists, prosthodontists, and sometimes neurologists or orthodontists, to address myofascial pain or occlusal issues [5].

A study by Guo et al. [10] used cone-beam computed tomography (CBCT) analysis to assess the relationship between periodontitis and TMJ, and found that severe periodontitis can alter TMJ space and condylar morphology, suggesting a mechanical link between the two conditions. However, a bidirectional Mendelian randomization study found no direct causal relationship, indicating that while periodontitis may aggravate TMD progression, the association is likely influenced by confounding factors, such as occlusal trauma or systemic inflammation [6].

Understanding the association between the severity of periodontitis and clinical TMJ symptoms is crucial for optimizing prosthodontic treatment outcomes. Therefore, this study aimed to explore the correlation between the severity of periodontitis and TMJ in patients requiring prosthodontic restoration, focusing on the clinical presentations and their implications for treatment planning. By integrating insights from periodontal, prosthodontic, and orofacial pain research, this study sought to provide evidence-based guidance for clinicians managing these interconnected conditions, ultimately improving patient comfort and functional restoration.

## Materials and methods

Study design and setting

This study employed a cross-sectional observational design to investigate the association between the severity of periodontitis and clinical TMJ symptoms in patients requiring prosthodontic restorations. It was conducted at Kothiwal Dental College and Research Centre, Moradabad, India, from March 2024 to March 2025. The study adhered to ethical guidelines, with approval obtained from the Institutional Ethical Review Board (KDCRC/IERB/01/2024/S04). Informed consent was obtained from all patients prior to enrolment. The study complied with the Declaration of Helsinki and ensured participant safety and confidentiality. The data were anonymized, stored securely, and accessible only to authorized researchers. Patients were informed of their right to withdraw at any time without affecting their clinical care.

Study population and sampling

The target population consisted of adult patients (aged 18-55 years) seeking treatment in the department. The inclusion criteria included a confirmed diagnosis of periodontitis and a clinical indication for prosthodontic restoration, such as crowns, bridges, or implants due to missing teeth. Exclusion criteria encompassed patients with systemic diseases affecting the TMJ (such as rheumatoid arthritis), acute periodontal infections, severe periodontitis with complete loss of dentition, or prior TMJ surgery.

A sample size calculation was performed using G*Power Software (Version 3.1.9.2, Heinrich-Heine University, Düsseldorf, Germany) to ensure adequate statistical power, targeting a minimum of 70 patients based on the prevalence estimate of periodontitis with TMD (p = 12%), at 80% power and a 5% confidence interval. Considering a 10% dropout rate, the present study was conducted on 80 patients. A stratified random sampling technique was used to ensure representation across varying severities of periodontitis.

Data collection

Data were collected through clinical examination, patient questionnaires, and diagnostic imaging. Periodontal status was assessed using clinical parameters such as probing depth, clinical attachment loss, and bleeding on probing, conducted by calibrated periodontists. TMJ symptoms, including patient-reported pain, jaw function limitations, and joint sounds, were noted. Standardized questionnaires were used to capture demographic data, medical history, and lifestyle factors (such as smoking and bruxism). CBCT scans were used to assess the condylar morphology and articular eminence angle in a subset of patients, ensuring comprehensive data on structural changes.

Assessment of periodontitis severity

The severity of periodontitis was classified based on the 2017 World Workshop on the Classification of Periodontal and Peri-Implant Diseases and Conditions [11]. Clinical measurements, including probing depth and attachment loss, were recorded at six sites per tooth using a periodontal probe. Radiographic evidence of bone loss was evaluated using CBCT tomography. The severity of periodontitis was categorized as initial periodontitis, moderate periodontitis, and severe periodontitis with loss of teeth, based on the extent of attachment loss and bone resorption, enabling the stratification of participants for analysis. All periodontal examinations were performed by two calibrated examiners, Harshita Pandey and Rupali Malik. 

Evaluation of TMJ symptoms

**Figure 1:**
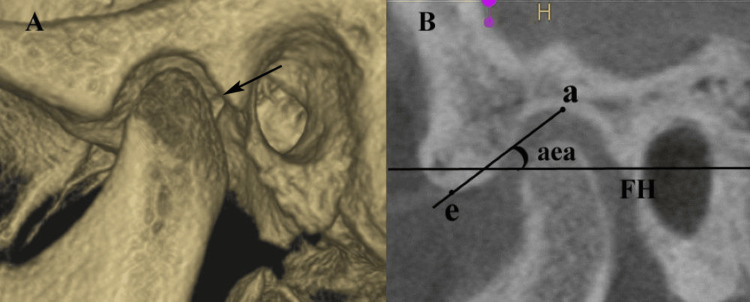
(A) CBCT image of temporomandibular joint and (B) articular eminence inclination angle (aea). This figure represents CBCT images of a study patient, included with appropriate informed consent. a: highest point on temporal fossa; e: highest point on articular eminence; FH: Frankfort Horizontal plane; CBCT: cone-beam computed tomography (sagittal view)

TMJ symptoms were assessed using the diagnostic criteria for TMDs (DC/TMD) protocol [12], which included clinical examinations and patient-reported outcomes. Trained examiners (Sumaiya Iman and Seema Gupta) evaluated the jaw movement range (deviation on opening the mouth), joint sounds (clicking or crepitus), and muscle or joint tenderness on palpation. The articular eminence inclination angle (aea) and condylar morphology were analyzed on the sagittal view of CBCT images by two calibrated examiners (Nosheen Riza and Premraj Jadhav), who were provided with coded CBCT scans for analysis to eliminate examiners' bias. The aea is formed between the Frankfort Horizontal plane (a plane passing through the lowermost point on the bony orbit and the uppermost point of the external auditory meatus) and the plane passing through the highest elevated points on the articular fossa (a) and articular eminence (e). The Fonseca Anamnestic Index (FAI) was used to assess the existence and intensity of TMD symptoms [13]. This index encompasses a total of 10 questions, each presenting three potential response categories (0 = no; 5 = occasionally; and 10 = affirmative), resulting in a cumulative score ranging from 0 to 100. This standardized approach ensured a reliable and reproducible assessment of TMD symptoms, facilitating a correlation with the severity of periodontitis (Figure 1). 

Statistical analysis

The data were analyzed by a statistician (Tripti Bhoi) using IBM SPSS Statistics for Windows, Version 23 (Released 2015; IBM Corp., Armonk, NY, USA). Descriptive statistics were used to summarize patient characteristics and clinical findings. The association between the severity of periodontitis and independent variables was analyzed using the chi-square test of association. The normality of the data was evaluated using the Shapiro-Wilk test, and data were found to be non-normally distributed. The parameters defining TMJ symptoms were compared between the periodontitis severity groups using the Kruskal-Wallis test, followed by post-hoc analysis using Dunn’s test. Principal component analysis (PCA) was used to assess the relationships between parameters affecting the TMJ. A significance level of p < 0.05 was used for all tests.

## Results

The analysis revealed significant associations between periodontal grade and TMJ symptoms. While sex distribution (p = 0.257) and joint sounds (p = 0.861) showed no statistically significant differences across groups, tenderness on palpation (p = 0.008) and condylar morphology (p = 0.001) were strongly associated with periodontitis severity. Tenderness was absent in healthy patients but was present in four (5%) patients with initial periodontitis, eight (10%) patients with moderate periodontitis, and eight (10%) patients with severe periodontitis, indicating progressive TMJ dysfunction. Condylar changes were most pronounced in patients with severe periodontitis. Degenerative changes, such as flattening, erosion, and osteophytes, were exclusive to the periodontitis groups, suggesting that occlusal instability and altered joint loading contributed to TMJ degeneration (Table 1).

**Table 1:** Analysis of association between periodontitis grades and temporomandibular joint (TMJ) symptoms using the chi-square test in a study sample (n = 80). *p-value < 0.05: significant. Data is presented in the form of frequency (n) and percentage (%).

Variables	Category	Healthy		Initial periodontitis		Moderate periodontitis		Severe periodontitis		Chi-square value	p-value
		n	%	n	%	n	%	n	%		
Sex	Male	14	18%	10	13%	8	10%	12	15%	4.04	0.257
	Female	6	8%	10	13%	12	15%	8	10%		
Joint sounds	No	14	18%	16	20%	14	18%	14	18%	0.75	0.861
	Yes	6	8%	4	5%	6	8%	6	8%		
Tenderness on palpation	No	20	25%	16	20%	12	15%	12	15%	11.73	0.008*
	Yes	0	0%	4	5%	8	10%	8	10%		
Condylar morphology	Normal	20	25%	14	18%	12	15%	6	8%	28.36	0.001*
	Flattening	0	0%	6	8%	4	5%	6	8%		
	Erosion	0	0%	0	0%	2	3%	4	5%		
	Osteophytes	0	0%	0	0%	2	3%	4	5%		

The Kruskal-Wallis test revealed highly significant differences (p < 0.001) in all TMJ parameters across the periodontal health groups. Mouth opening progressively decreased from healthy (34.20 ± 1.70 mm) to severe periodontitis (27.20 ± 2.63 mm), reflecting functional impairment. Deviation during opening worsened significantly (healthy: 2.20 ± 0.77; severe periodontitis: 5.50 ± 1.40), indicating asymmetric joint movement. Articular eminence inclination steepened with disease severity (healthy: 43.20 ± 6.79; severe periodontitis: 60.20 ± 2.97), suggesting biomechanical adaptation to occlusal instability. Most notably, the FAI (pain/dysfunction) increased from 6.40 ± 2.16 (healthy) to 19.80 ± 3.81 (severe periodontitis), confirming that symptom burden correlated with periodontal destruction. These findings demonstrated that TMJ dysfunction worsened proportionally with periodontitis severity, with a significant decline in joint mobility, stability, and comfort (Table 2). 

**Table 2:** Comparison of temporomandibular joint (TMJ) parameters between study groups by Kruskal-Wallis test. *p-value < 0.05: significant. Data is presented in the form of mean ± standard deviation (SD).

Variables	Healthy	Initial periodontitis	Moderate periodontitis	Severe periodontitis	Chi-square value	p-value
	Mean ± SD	Mean ± SD	Mean ± SD	Mean ± SD		
Mouth opening in mm	34.20 ± 1.70	34.20 ± 1.51	30.80 ± 1.70	27.20 ± 2.63	57.44	0.001*
Deviation of mouth opening in degrees	2.20 ± 0.77	2.50 ± 1.47	3.70 ± 1.13	5.50 ± 1.40	41.83	0.001*
Articular eminence inclination in degrees	43.20 ± 6.79	50.30 ± 3.70	57.00 ± 3.18	60.20 ± 2.97	59.11	0.001*
Anamnestic index	6.40 ± 2.16	11.20 ± 2.55	14.50 ± 1.85	19.80 ± 3.81	64.53	0.001*

The post-hoc test revealed significant differences (p < 0.05) in the TMJ parameters between the periodontal groups. The severe periodontitis group showed the most pronounced deviations, with significantly reduced mouth opening, greater deviation, steeper articular eminence inclination, and higher FAI scores compared to healthy controls. Mouth opening and articular inclination were similar in the healthy and initial periodontitis groups. The moderate periodontitis group differed significantly from the other groups (p < 0.016). Notably, pain and dysfunction (FAI) progressively escalated across all stages. These results confirmed that TMJ degeneration was most severe in severe periodontitis, but functional and structural changes began as early as in moderate periodontitis (Table 3).

**Table 3:** Post-hoc analysis with Dunn's test for temporomandibular joint (TMJ) parameters between study groups. *p-value < 0.05: significant.

Pairwise comparisons	Mouth opening		Deviation of mouth opening		Articular eminence		Anamnestic index	
	z-value	p-value	z-value	p-value	z-value	p-value	z-value	p-value
Severe periodontitis - Healthy	-6.242	0.001*	5.795	0.001*	5.795	0.001*	7.680	0.001*
Severe periodontitis - Initial periodontitis	-6.256	0.001*	5.201	0.001*	5.201	0.001*	4.775	0.001*
Severe periodontitis - Moderate periodontitis	-2.274	0.023*	2.780	0.005*	2.780	0.005*	2.442	0.015*
Healthy - Initial periodontitis	-0.014	0.989	-0.595	0.552	-0.595	0.552	-2.906	0.004*
Healthy - Moderate periodontitis	3.968	0.001*	-3.015	0.003*	-3.015	0.003*	-5.238	0.001*
Initial periodontitis - Moderate periodontitis	3.982	0.001*	-2.420	0.016*	-2.420	0.016*	-2.333	0.020*

**Figure 2:**
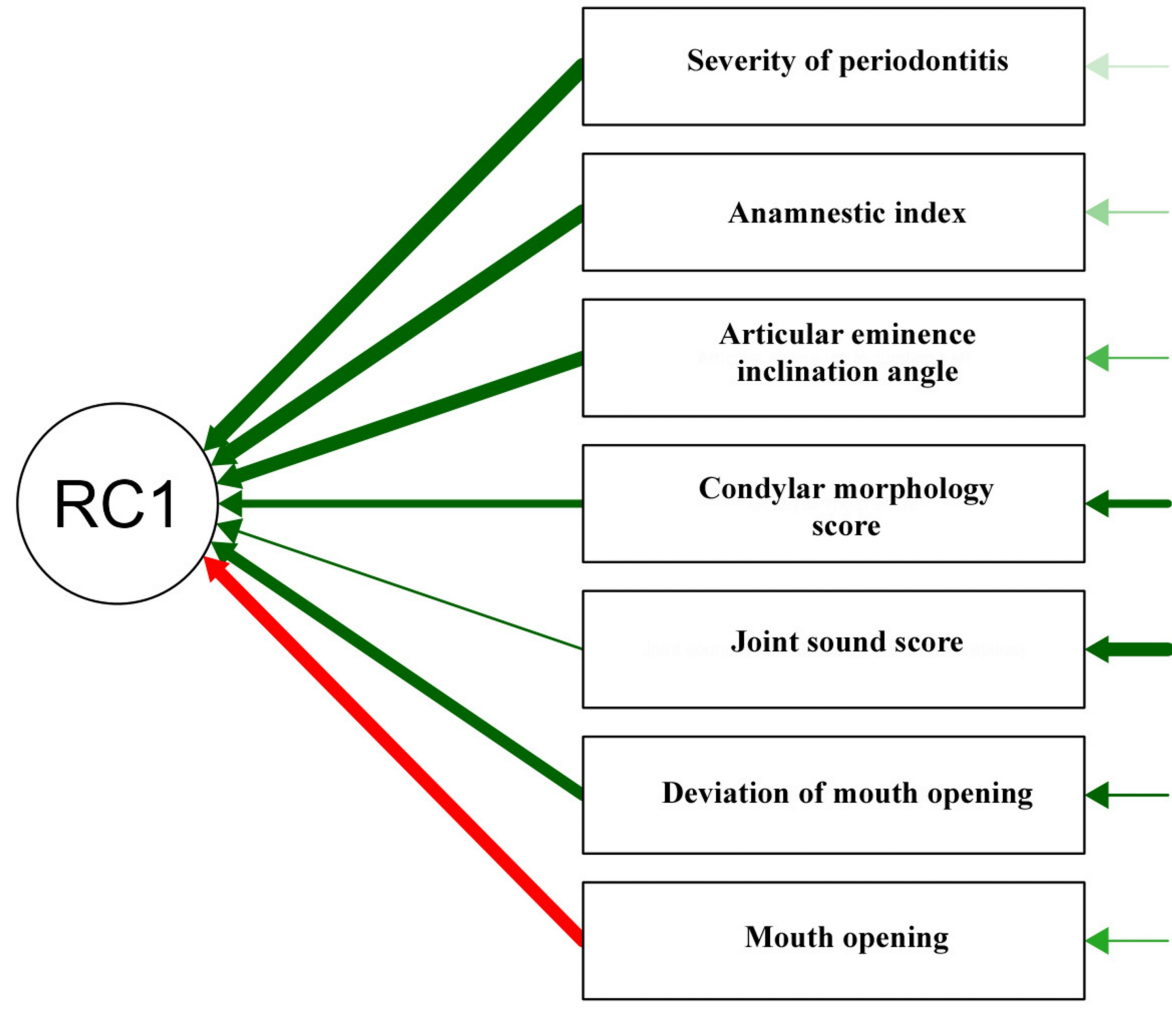
Principal component analysis for various factors. The red arrow denotes a negative relation between two components, the green arrow denotes a positive relation between two components, and the thickness of the green arrow denotes the loading value. This figure is derived from the data of the study. RC1: rotated component 1

PCA revealed that RC1 (first component) explained 72.3% of the variance, strongly correlated with periodontitis severity (loading = 0.96) and TMJ dysfunction (FAI = 0.92). Structural changes, such as steeper articular eminence (0.85) and reduced mouth opening (-0.81), loaded highly, while condylar morphology (0.62) and joint sounds (0.42) contributed moderately. The high uniqueness values for joint sounds (0.826) and condylar morphology (0.622) indicated that these parameters were less explained by RC1. The analysis confirmed that periodontitis severity drove TMJ degeneration, with RC1 representing a "TMJ-pathology continuum," linking occlusal damage to joint dysfunction (Table 4 and Figure 2).

**Table 4:** Principal component analysis with component loading. RC1: rotated component 1

Parameters	RC1	Uniqueness
Severity of periodontitis	0.96	0.078
Anamnestic index	0.92	0.163
Articular eminence inclination	0.85	0.279
Mouth opening	-0.81	0.34
Deviation of mouth opening	0.76	0.423
Condylar morphology	0.62	0.622
Joint sounds	0.42	0.826

## Discussion

The findings of this study provided compelling evidence for a significant association between the severity of periodontitis and clinical TMJ symptoms in patients requiring prosthodontic restoration. The observed correlations between periodontitis severity and TMJ dysfunction, particularly in parameters such as tenderness on palpation, condylar morphology, mouth opening, and the FAI, underscore the importance of a multidisciplinary approach to treatment planning.

The results of this study indicated that TMJ dysfunction worsened proportionally with the severity of periodontitis, with significant differences in functional and structural parameters across periodontal health groups. Similar findings have been reported by Guo et al. [10]. Notably, tenderness on palpation (p = 0.008) and condylar morphology (p = 0.001) were strongly associated with the severity of periodontitis, with degenerative changes, such as flattening, erosion, and osteophytes, observed exclusively in the periodontitis groups. These findings suggest that periodontal bone loss and associated occlusal instability may contribute to altered joint loading and the precipitation of TMJ degeneration. The progressive decline in mouth opening (from 34.2 ± 1.7 mm in healthy controls to 27.2 ± 2.63 mm in severe periodontitis) and increased deviation during opening (from 2.20 ± 0.77 to 5.50 ± 1.40) further highlighted the functional impairments linked to periodontal disease severity. These changes have direct implications for prosthodontic treatment, as occlusal instability and restricted jaw mobility can complicate the design and success of restorations such as crowns, bridges, or implants.

The increase of the FAI scores (from 6.40 ± 2.16 in healthy controls to 19.8 ± 3.81 in severe periodontitis) reflected a significant symptom burden, including pain and dysfunction, which aligns with previous studies linking periodontal disease to orofacial pain [1,13]. This finding emphasizes the need for clinicians to assess TMJ symptoms in patients with periodontitis before initiating prosthodontic treatment. For instance, patients with severe periodontitis may require targeted interventions, such as occlusal splints or physical therapy, to stabilize the TMJ before proceeding with restorations. Moreover, the steeper articular eminence inclination observed in severe periodontitis (60.20 ± 2.97 vs. 43.20 ± 6.79 in healthy controls) suggested biomechanical adaptations to occlusal changes, which might necessitate adjustments in prosthetic design to restore optimal occlusal relationships.

The association between periodontitis and TMJ symptoms likely stems from a combination of biomechanical, inflammatory, and behavioral factors. Periodontitis leads to alveolar bone loss, which compromises tooth support and alters occlusal relationships [5]. As periodontitis advances, discomfort resulting from inflammation may compel patients to engage in unilateral mastication, which can lead to an abnormal occlusal relationship or potentially result in occlusal trauma [14]. This occlusal instability can increase mechanical stress on the TMJ, leading to degenerative changes, such as condylar flattening and osteophyte formation, as observed in this study. PCA also supported this hypothesis, with RC1 explaining 72.3% of the variance and showing strong correlations between periodontitis severity (loading = 0.96), TMJ dysfunction (FAI = 0.92), and structural changes, such as articular eminence inclination (0.85) and reduced mouth opening (-0.81). These findings suggested a "TMJ-pathology continuum," in which periodontal destruction drove occlusal and joint dysfunction [5,15].

Inflammation may also play a role in this process. Periodontitis is characterized by a sustained inflammatory response, with elevated levels of pro-inflammatory cytokines, such as interleukin-1β and tumor necrosis factor-α [16]. These cytokines may contribute to synovial inflammation in the TMJ and exacerbate symptoms such as tenderness and pain [17]. Although this study did not measure inflammatory markers, the significant association between tenderness on palpation and periodontitis severity supports the hypothesis of an inflammatory link. Future studies that incorporate biomarkers can further elucidate this mechanism.

Behavioral factors, such as bruxism, may also mediate the relationship between periodontitis and TMJ symptoms [18]. Bruxism, a known risk factor for TMD, can exacerbate occlusal instability in patients with periodontal bone loss, leading to increased joint loading and degeneration [18]. While the study controlled for some lifestyle factors, such as smoking, the influence of bruxism was not fully explored because of reliance on patient-reported data. This represents an area for future investigation, as objective measures of bruxism (such as polysomnography) could strengthen the understanding of its role.

The findings of our study align with those of previous research demonstrating a link between periodontal disease and TMD [5,10]. However, our findings contradict those of a study by Wang et al. [6], where no association was reported between periodontitis and TMDs. The disparity in the results could have been due to differences in the methodology. A study by LeResche et al. [19] reported that patients with periodontal disease were more likely to experience TMD symptoms, particularly pain and restricted jaw movements. However, our study advances the field by using a standardized protocol (DC/TMD) and CBCT imaging to provide a comprehensive assessment of TMJ symptoms and morphology, thus addressing the limitations of earlier studies that relied on less rigorous diagnostic criteria.

The absence of significant differences in joint sounds (p = 0.861) across periodontal groups is consistent with prior research, indicating that joint sounds, such as clicking, are less specific to TMD severity and may occur in healthy individuals [20]. In contrast, the strong association between condylar morphology and periodontitis severity (p = 0.001) corroborates studies suggesting that occlusal changes contribute to TMJ degeneration [5]. The PCA findings further supported this, highlighting that structural and functional parameters were more closely tied to periodontitis severity than joint sounds, which had high uniqueness values (0.826) in the analysis.

Clinical implications for prosthodontic treatment

The interplay between periodontitis and TMJ symptoms has significant implications in prosthodontic treatment planning. Patients with moderate-to-severe periodontitis may require a phased approach to address periodontal health and TMJ stability before proceeding with restorations. For example, periodontal therapy, such as scaling and root planing, may reduce inflammation and stabilize occlusion, potentially alleviating TMJ symptoms. Similarly, occlusal adjustments or splint therapy may be necessary to manage TMD symptoms and ensure the longevity of prosthetic restorations.

The study’s findings also underscore the importance of interdisciplinary collaboration among periodontists, prosthodontists, and orofacial pain specialists. By integrating periodontal and TMJ assessments into the treatment workflow, clinicians can develop personalized and effective treatment plans. For instance, patients with severe periodontitis and pronounced TMJ symptoms may benefit from CBCT-guided prosthetic planning, to account for the altered condylar morphology and articular eminence inclination.

Limitations of the study

Despite its strengths, this study had several limitations. This cross-sectional design precluded the establishment of causality between periodontitis and TMJ symptoms. Longitudinal studies are needed to determine whether periodontal treatment can mitigate TMJ dysfunction, or whether TMD interventions improve periodontal outcomes. Additionally, reliance on patient-reported outcomes for some variables, such as bruxism, might have introduced recall bias. Objective measures, such as electromyography for muscle activity or polysomnography for bruxism, could enhance the accuracy of these assessments. The sample size (n = 80), although statistically adequate, might not fully capture the heterogeneity of TMD presentations, particularly in patients with milder forms of periodontitis. Furthermore, the study was conducted in a single centre, which might limit generalizability to other populations with different demographic or clinical profiles. The exclusion of patients with systemic diseases affecting the TMJ, such as rheumatoid arthritis, while isolating the effects of periodontitis, might have overlooked potential interactions between systemic conditions and TMJ symptoms.

Future directions

Future studies should focus on longitudinal designs to explore the temporal relationship between periodontitis progression and TMJ degeneration. Incorporating inflammatory biomarkers can clarify the role of systemic inflammation in mediating this association. Additionally, studies investigating the efficacy of combined periodontal and TMD interventions, such as periodontal therapy paired with occlusal splints, could provide practical guidance to clinicians. The use of advanced imaging techniques, such as dynamic magnetic resonance imaging (MRI), could further elucidate biomechanical changes in the TMJ associated with periodontal disease.

## Conclusions

This study established a significant association between the severity of periodontitis and clinical TMJ symptoms in patients requiring prosthodontic restoration. The findings highlighted that, as periodontitis severity increased, TMJ dysfunction worsened, characterized by a progressive decline in joint mobility, increased pain, and structural changes in the TMJ. These associations underscore the need for integrated periodontal and TMJ assessments in prosthodontic treatment planning to effectively address occlusal instability and joint dysfunction. A multidisciplinary approach, which combines periodontal therapy, TMJ management, and tailored prosthetic interventions, is essential to optimize treatment outcomes, enhance patient comfort, and achieve successful functional restoration. The results support comprehensive clinical evaluations to ensure personalized care for patients with coexisting periodontitis and TMJ symptoms.
